# High sulfur and nitrogen foods are the main triggers of parosmia

**DOI:** 10.1007/s00405-026-10270-4

**Published:** 2026-05-21

**Authors:** Leonardo de Moura Volpi, Eduardo Takashi Hirata, Luiza Helena Chuque Medina, Juliana Pascutti Sant’Ana, Bruno Fernandes de Siqueira, Marcel Menon Miyake

**Affiliations:** 1 Otorhinolaryngology Department, Irmandade da Santa Casa de Misericórdia de São Paulo, São Paulo, SP 01221-020 Brazil; 2 Fundação Firmenich, Kaiseraugst, Switzerland

**Keywords:** Olfactory disorders, Parosmia, Olfaction, Quality of life, Chemical biology

## Abstract

**Purpose:**

Parosmia is a condition associated with a significant impact on quality of life. Although its pathophysiology remains poorly understood, certain aromas and fragrances are frequently reported as triggers. This study aimed to identify the primary triggers of parosmia and analyze their molecular similarities.

**Methods:**

This cross-sectional study included individuals with current complaints of parosmia. Data were prospectively collected via an online survey that assessed the most commonly reported triggers and associated perceptions. A perfumer analyzed the molecular characteristics of these substances.

**Results:**

A total of 102 individuals with parosmia were included. The most frequently reported triggers were onion (68%), garlic (62.9%), coffee (49.5%), and beef (48.5%). These substances share sulfur-containing, nitrogenous, and thioester components, characterized by high olfactory potency and sensory notes described as “rotten,” “sulfurous,” and “roasted/burnt.” The most associated perceptions were sewage (71%) and burnt (37.6%).

**Conclusion:**

The primary triggers of parosmia appear to share significant molecular similarities. This knowledge may contribute to a better understanding of the pathophysiology of parosmia and serve as a preliminary step toward developing new therapeutic approaches for this disorder.

**Supplementary Information:**

The online version contains supplementary material available at 10.1007/s00405-026-10270-4.

## Introduction

Parosmia is an olfactory disorder characterized by a distorted and typically unpleasant perception of odors. The quality of life of affected individuals is significantly affected, as olfaction plays a crucial role in daily activities, including food consumption, safety, and social interactions. The inability to accurately identify and interpret smells can lead to nutritional challenges, environmental hazards, and emotional distress [1–3].

Despite growing recognition of parosmia—particularly given its increased prevalence following SARS-CoV-2 infection—its pathophysiology remains poorly understood. The complex mechanisms underlying olfactory distortion have hindered the development of accurate diagnostic tools and effective therapeutic strategies. Several theories have been proposed to explain parosmia, ranging from aberrant axonal regeneration of olfactory neurons [4, 5] to central nervous system involvement [6]. However, few studies have examined the condition from the perspective of the odorant molecules that elicit distorted perceptions.

Certain everyday substances, such as onion, meat, coffee, and toothpaste, frequently emerge as parosmia triggers, yet the molecular basis of this association remains poorly defined [1]. A promising approach was demonstrated by Parker et al. [7], who used chromatography to analyze the chemical constituents of coffee and identified specific compounds that triggered unpleasant olfactory experiences in patients with parosmia. Their findings suggested that key molecular triggers share similar structural features and possess low olfactory detection thresholds.

Identifying the specific odorant molecules associated with parosmia could provide valuable insights into the disorder’s pathophysiology and potentially guide the development of therapeutic products to mitigate its effects. Accordingly, the present study sought to determine the primary triggers of parosmia through an online questionnaire and to investigate the molecular similarities among these odorants.

## Methods

This was a cross-sectional observational study that evaluated patients presenting with parosmia, with a focus on identifying the most frequently reported odorant triggers. Subsequently, a professional perfumer analyzed the molecular characteristics of these substances and explored potential similarities among them. Data were collected and stored using Google Forms. All participants were informed of the risks and benefits of the study and provided electronic consent through the same platform. The study was approved by the Research Ethics Committee of Faculdade de Ciências Médicas da Santa Casa de São Paulo, in Sao Paulo, Brazil.

### Participants

Participants of both sexes aged 18 to 75 with a current complaint of parosmia were included. Parosmia was defined as a self-reported distortion in the perception of smell and/or taste. Patients reporting exclusively hyposmia or anosmia, with no symptoms of parosmia, were not included. Exclusion criteria included individuals under 18 years old, pregnant or breastfeeding women, those using immunosuppressive medications, and patients who had fully recovered from parosmia or reported only quantitative olfactory disorders.

Participants were recruited throughout November 2022 from the “Sinonasal Diseases” outpatient clinics of the Department of Otolaryngology at the Irmandade da Santa Casa de Misericórdia de São Paulo, as well as through announcements in parosmia support groups on social media platforms such as WhatsApp and Facebook.

### Assessment of results

All participants completed a two-part online questionnaire. The first section collected demographic and clinical data, with an emphasis on identifying parosmia triggers. Data points included age, sex, ethnicity, history of sinonasal disease, and smoking status. Clinical characteristics of parosmia—specifically onset velocity (sudden vs. progressive), duration since onset, clinical course (improvement vs. deterioration), and reported etiology (COVID-19, vaccination, head trauma, or drug-induced)—were recorded. Participants identified specific triggers and described their responses. Predefined trigger options were provided based on established literature (e.g., onion, garlic, coffee, meat, fried odors, and perfumes), with an option for open-ended additions. Finally, participants were asked which odors they experienced from a predefined list (sewage, burnt, rotten egg, citrus, wet sock, garlic, onion, raw meat), based on the authors’ experience or additional patient reports.

The second section assessed subjective olfactory dysfunction using the Questionnaire of Olfactory Disorders (QOD) [8–10], adapted from the validated Brazilian Portuguese version [11]. To the authors’ knowledge, the QOD is the only patient-reported questionnaire specifically developed to qualitatively evaluate olfactory disorders. It was originally created by Frasnelli and Hummel [8] and includes 52 items divided into three domains: 39 “negative” statements, 5 “positive” statements (PS), and 8 “socially desired” statements (DS). Bernardino et al. [11] validated this questionnaire for Brazilian subjects and improved its internal consistency by reducing it to a 29-item version. This version, employed in the present study, is divided into three domains: 1) parosmia-related statements, 2) quality of life statements, and 3) sincerity statements. Responses were recorded on a scale ranging from “I totally agree” to “Partially disagree” to “Totally disagree.” The first domain concerns the presence of parosmia symptoms, while the second assesses the severity of parosmia and its impact on patients’ daily lives. The final domain was designed to detect whether patients gave socially desirable responses. The full QOD questionnaire is available in the Electronic Materials.

After pinpointing the most frequently reported odorant triggers, a professional perfumer conducted a descriptive molecular analysis of the key aromatic compounds in these substances, drawing on specialized literature and emphasizing possible similarities among them.

## Results

**Fig. 1:**
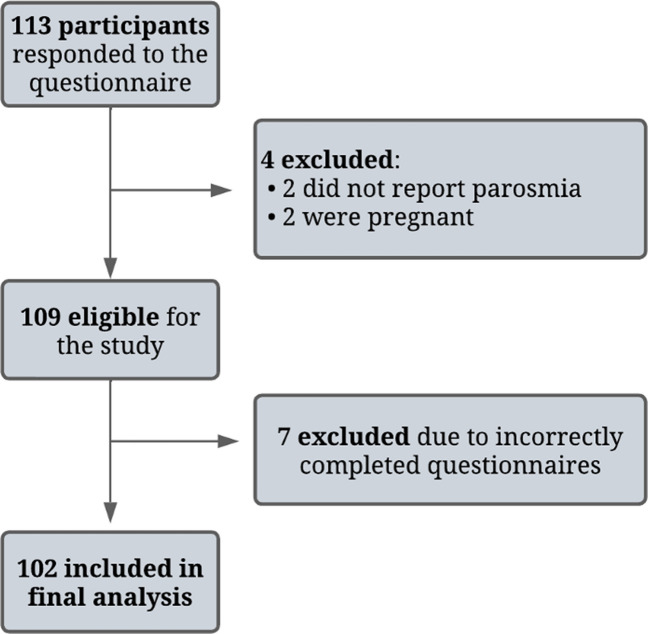
Study population

A total of 113 individuals were recruited and completed the questionnaire. During the one-month inclusion period, two participants were recruited from the outpatient clinic, and 111 patients via online support groups. Four were excluded: two did not report active parosmia at the time of the study, and two were pregnant. Among the 109 eligible participants, 7 were excluded for incomplete or invalid questionnaire responses, yielding a final sample of 102 (Fig. 1).

The average age of participants was 35.6 ± 9.56 years. There was a higher proportion of female participants (89.2%) and individuals of Caucasian ethnicity (63.7%). The mean duration since parosmia onset was 18.9 ± 5.8 months. The most common cause was COVID-19 infection (95.1%). In most cases, symptom onset was sudden (65.7%) and was followed by gradual partial improvement (62.7%) (Table 1).

**Table 1:** Baseline characteristics

Clinical and Demographic		*N* = 102 (%)
Age (average ± SD)		35,5 ± 9,6
Sex (%)	Female	88 (89,2%)
	Male	9 (10,8%)
Ethnicity (%)	Caucasian	65 (63,7%)
	Black	6 (5,9%)
	Mixed-race	27 (26,5%)
	Asian	2 (2%)
	Others	2 (2%)
Nasal disease (%)	Rhinitis	34 (33,3%)
	Deviated septum	20 (19,6%)
	Chronic rhinossinusitis	9 (8,8%)
Smoking (%)		4 (4,2%)
Parosmia		
Onset (%)	Sudden	67 (65,7%)
	Progressive	35 (34,3%)
Mean time since onset (average ± SD)		18,9 ± 5,8
Course (%)	Improvement	64 (62,7%)
	Stable	27 (26,5%)
	Worsening	11 (10,8%)
Reported cause (%)	COVID-19	97 (95,1%)
	COVID-19 vaccination	3 (2,9%)
	Traumatic brain injury	1 (1%)
	Drug-induced	1 (1%)

**Fig. 2:**
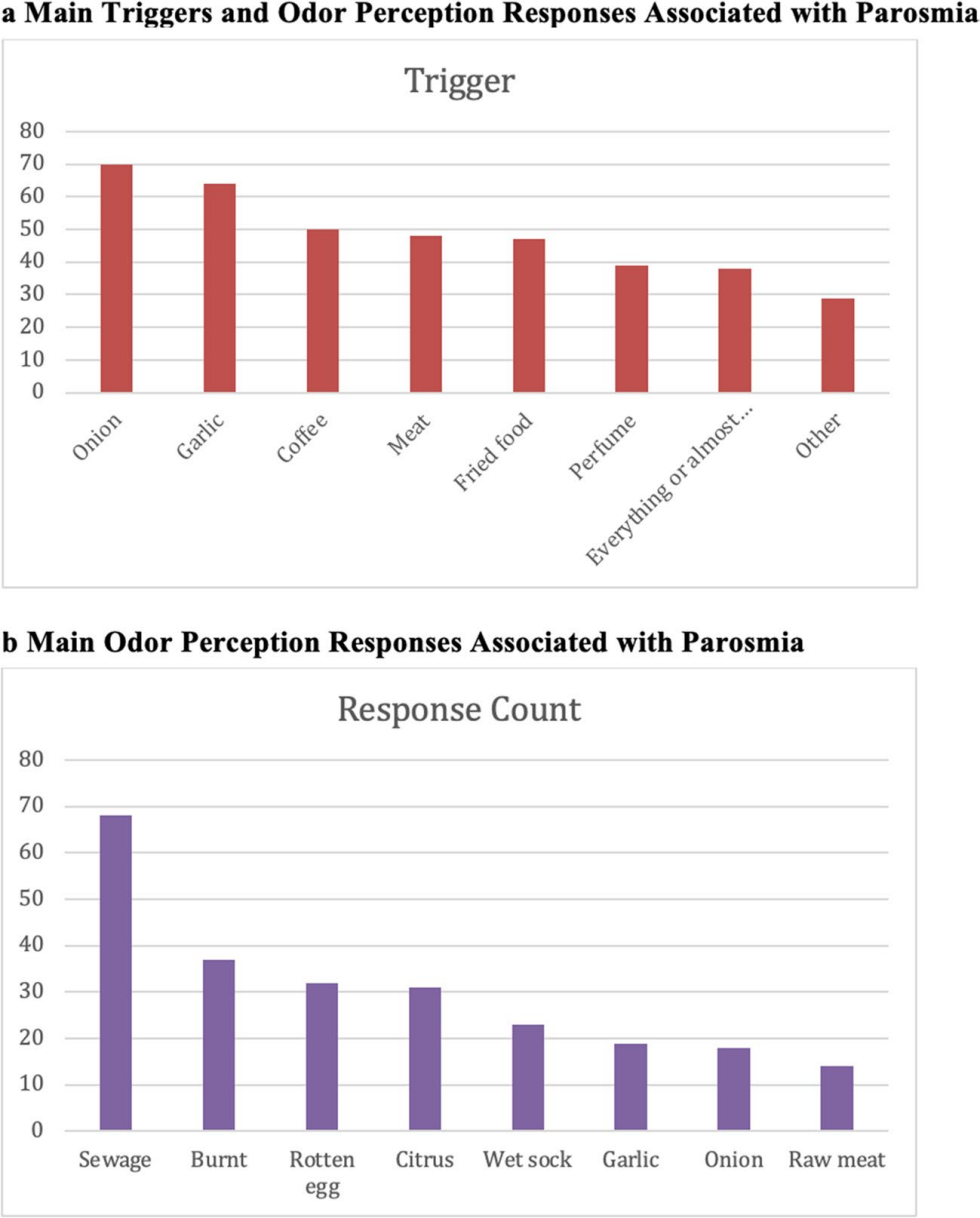
Main trigger and order perception responses associated with Parosmia. b Main order perception responses associated with Parosmia

The most frequently reported parosmia triggers were onion (68.6%) and garlic (62.7%), followed by coffee (49.5%), meat (47.1%), fried food odors (46.1%), and perfumes (38.4%). About one-third of participants reported perceiving an unpleasant odor in response to nearly all smells (Fig. 2a). Only 29 participants (28.4%) identified other foods or odors as triggers. Other reported triggers included eggs, milk and dairy, fruits, and gasoline.

Concerning the qualitative nature of the distorted odors, the most frequently reported smell was sewage (69.4%), followed by burnt odor (37.8%), rotten eggs (32.7%), citrus-like odor (31.6%), wet socks (23.5%), garlic (19.4%), onion (18.3%), and raw meat (14.3%) (Fig. 2b).

The mean total QOD score in this study sample was 58.8 ± 11.8 out of a maximum of 87 points (Table 2). The Parosmia Domain revealed severe symptoms, with a mean score of 10.2 ± 1.76 out of 12 points. The Quality of Life Domain yielded a mean score of 49.8 ± 9.9 out of 57 points, indicating a substantial impact on patients’ daily functioning. The Sincerity Domain showed low response bias, with a mean score of 7.7 ± 2.7, which is close to the midpoint of the total possible score (18 points). The complete distribution of responses for each QOD item is available in Online Resource 1.

**Table 2:** Questionnaire of olfactory disorders (QOD) scores by statement

QOD Statement	Average ± SD	Maximum possible result
Parosmia	10.2 ± 1.8	12
Sincerity	7.8 ± 2.7	18
Quality of Life	40.8 ± 9.9	57
Total	58.8 ± 11.8	87

**Fig. 3:**
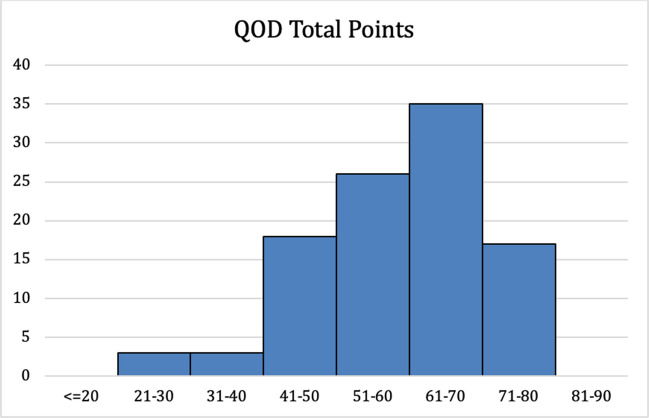
Distribution of patients across Questionnaire of Olfactory Disorders (QOD) severity strata

Total QOD scores were stratified into 10-point intervals to classify parosmia severity. The distribution of QOD scores is shown in Fig. 3. Approximately one-third of the sample (35 participants) scored between 61 and 70, representing the most frequent score range. The distribution demonstrated a clear rightward skew, with only 24 patients falling within the lower four strata (0–50 points). The remaining 78 participants scored between 51 and 87 points. No participants scored below 20 or above 81.

### Garlic

Garlic contains approximately 0.1% to 0.25% essential oil, which is enzymatically formed when the cloves are crushed, cut, or rehydrated. This oil is composed primarily of sulfur-containing compounds, including approximately 60% diallyl disulfide, 20% diallyl trisulfide, and 6% allyl propyl disulfide. Allicin (diallyl disulfide) is the key compound responsible for garlic’s pungent aroma (Table 3).

**Table 3:**
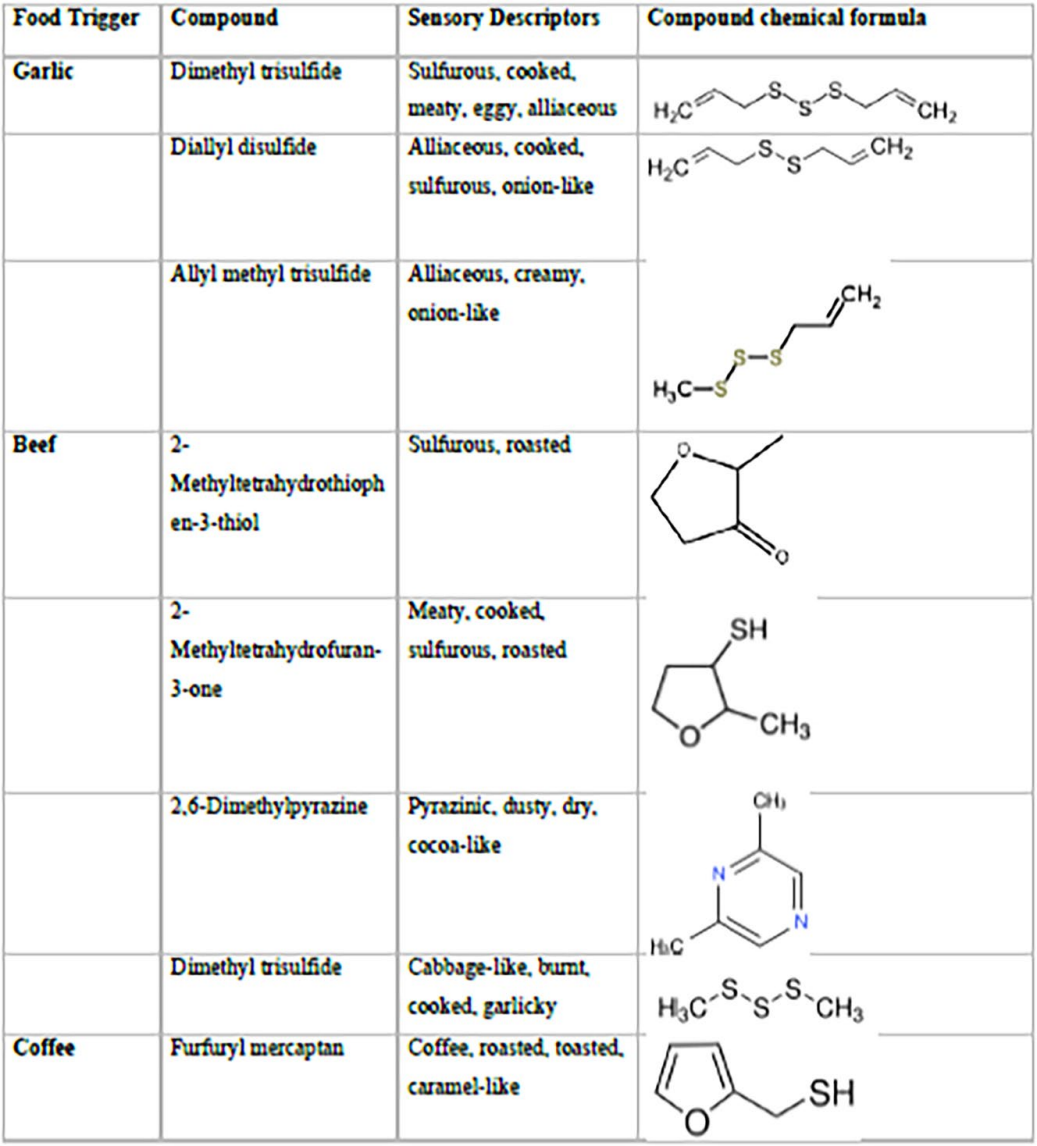
Key odorant molecules implicated in parosmia triggers

Intact garlic has virtually no odor, but once cut or crushed, it releases a strong, pungent smell. This occurs because the enzyme alliinase in fresh garlic acts on alliin to produce allicin, which then rapidly decomposes into diallyl disulfide, yielding the characteristic sharp sulfurous odor. Pungency tends to diminish during cooking or roasting. Cooking softens the flavor, while roasting imparts a mellow, balanced aroma with nutty undertones. Dried garlic, particularly white-skinned varieties, has a notably strong and persistent odor and taste. In contrast, roasted garlic develops a mildly sweet and delicate flavor [12].

### Coffee

Approximately 850 volatile compounds have been identified in coffee flavor. However, only a small subset—roughly 40 volatile compounds with low odor thresholds and/or high concentrations—are considered the primary contributors to coffee aroma. These key volatiles include carbonyls, furans, alicyclic sulfur compounds, aromatic benzenoids, and various heterocyclic compounds.

Roasting initiates a cascade of chemical reactions within coffee beans, leading to the formation of aromatic volatiles that define the coffee aroma profile. These reactions include the Maillard reaction (a non-enzymatic browning process involving nitrogen-containing compounds and carbohydrates under heat), Strecker degradation (involving amino acids and carbonyl compounds in aqueous environments), and the thermal degradation of individual amino acids, trigonelline, sugars, phenolic acids, and lipids.

Among the aromatic volatiles most relevant to the characterization of coffee flavor, sulfur-containing compounds and pyrazines are particularly pertinent to the current study. Furfuryl mercaptan is arguably the most critical compound, even at extremely low concentrations. This molecule—also found in meat, caramel, and seafood—contributes distinct roasted, burnt, and caramel-like notes to coffee's aroma.

Pyrazines are also highly influential, contributing with brown, roasted, and caramel notes and playing a key role in flavor profiles associated with meat, chocolate, bacon, and other foods [12] (Table 3).

### Beef

The flavor of beef arises from several hundred volatile compounds, although only a limited number are responsible for its characteristic aroma. These compounds are formed through complex, multidirectional reactions involving non-volatile precursors naturally present in raw meat—such as amino acids, reducing sugars, saturated fats, and vitamins—under specific conditions of temperature, pressure, pH, and time. Key pathways include the Maillard reaction, lipid oxidation, enzymatic degradation followed by Maillard transformation, and thiamine breakdown.

Flavor development can vary considerably based on the raw material (e.g., breed, sex, diet, and age of the animal), slaughtering conditions, storage time and environment, muscle type, applied additives, and the course of thermal processing.

Temperature is a critical factor in initiating these reactions. Depending on the cooking method, beef's flavor profile can vary according to consumer preference. For instance, grilling tends to produce more pronounced “charred” or “roasted” notes, whereas boiling or pan-frying may yield “fatty” or “toasted” notes. Although the preparation method influences specific aromatic nuances, certain chemical families consistently contribute to the core beef flavor. Among these, sulfur-containing compounds, nitrogenous compounds, and thioesters are the major contributors to the distinct aroma profile of cooked beef [12] (Table 3).

## Discussion

Parosmia is an olfactory disorder that has long challenged medical understanding and has a profound impact on patients’ quality of life [1]. In this study, approximately one-third of participants reported experiencing parosmia in response to most foods. Although the subjective nature of olfactory complaints must be recognized, these findings strongly indicate that parosmia substantially affects daily life by impairing the ability to enjoy odors during routine activities.

The application of a validated olfactory-specific quality of life instrument (QOD) further supported this finding, demonstrating elevated scores in both the parosmia severity and quality of life domains. The association between symptom severity and quality of life reduction is anticipated, as olfactory distortions frequently occur unpredictably, rendering social interactions and dining experiences challenging—and occasionally unpleasant [2, 13, 14].

The findings of this study align with previous reports [4, 7], indicating that most patients with parosmia perceive odors in a significantly negative way, frequently employing descriptors such as “sewage” and “burnt,” which underscores the distressing and unpleasant nature of these olfactory distortions.

The primary food triggers identified in this study were onion (68.4%), garlic (62.7%), coffee (49.5%), and meat (48.5%). These foods share key aromatic chemical components, primarily sulfur, nitrogen, phenol, pyrazine, thioesters, and furan. These odor-active compounds are highly concentrated and detectable at extremely low thresholds—on the order of 10⁻^12^. They are exceptionally potent, and from a sensory perspective, are often associated with putrid, sulfurous, and roasted or burnt notes [12].

Similar observations were reported by Parker et al. ([7]), who utilized gas chromatography–olfactometry to identify the key odorant compounds in coffee that trigger parosmia in affected individuals. In line with the present study, the main chemical groups involved in parosmia included thiols, pyrazines, and disulfides—rich in sulfur- and nitrogen-containing components. Additionally, the authors noted that these compounds have exceptionally high olfactory potency and very low odor detection thresholds, meaning they are detectable at remarkably low concentrations.

Identifying these specific chemical triggers represents a significant step forward in understanding parosmia's pathophysiology. Although several hypotheses have been suggested, the underlying mechanisms of parosmia remain poorly understood. Some evidence points to central nervous system dysfunction, as indicated by olfactory bulb volume reduction in patients with olfactory disorders [6]. However, subsequent studies have also identified changes in brain regions associated with the emotional valence of odors in patients with parosmia [4].

Furthermore, recent evidence of extensive damage to the olfactory epithelium caused by COVID-19 has led to the hypothesis of aberrant reinnervation of olfactory neurons during the regenerative process [5]. While both central and peripheral mechanisms may coexist, they do not fully explain the fluctuating nature of hyposmia or the intense, specific responses to certain odorants observed in this study. These findings suggest that the sensory epithelium of the olfactory system might play a more significant role in this dysfunction than previously recognized.

Identifying the chemical compounds in key parosmia-triggering foods is a crucial first step in developing olfactory tests specific to this condition. These advancements could also facilitate the creation of patient counseling tools that explain which foods are most likely to induce distorted smell and taste perceptions.

Collaboration between clinicians and fragrance or flavor specialists in the food and perfumery industries could also open new pathways for developing alternative treatments for olfactory disorders. These industries regularly confront the challenge of masking unpleasant odors and improving sensory experiences. Similarly, if foods that trigger parosmia share chemical similarities, it might be possible to create products—such as sauces or condiments—that, when added to these foods, can reduce the unpleasant sensory experience, thereby lessening the impact on patients’ quality of life.

Nevertheless, it is important to acknowledge the limitations of this study. While the results offer a solid foundation for future research, the complexity of olfactory perception and the pathophysiology of parosmia suggest a multifactorial process. This study relied on self-reported data, which may introduce bias and inter-individual variability during the interpretion of the results. The sampling method also presented limitations, as participants were recruited mostly through voluntary responses to online questionnaires within patient support groups, potentially leading to an overrepresentation of more severe cases. The significant sex imbalance in the sample should be addressed in future studies to reduce selection bias. The perfumer characterized the odorant molecules by identifying the most prevalent chemical compounds found in the reported triggers, based on established literature. Future research should prioritize analytical chemical approaches (such as compound isolation, as performed by Parker et al.) and present them in person. Such methodologies would facilitate a more precise identification of the specific odorant molecules responsible for parosmic distortions. Finally, a more comprehensive investigation into comorbid parosmia and quantitative olfactory dysfunction (hyposmia/anosmia) is warranted.

In summary, this study identified a strong link between parosmia and foods rich in sulfur- and nitrogen-containing compounds, highlighting shared molecular features among the most common triggers. These findings not only improve our understanding of parosmia’s underlying mechanisms but also provide a solid foundation for future research aimed at creating new treatment options.

## Conclusion

Onion, garlic, coffee, and meat—foods rich in potent sulfur- and nitrogen-containing aromatic compounds—were identified as the primary triggers of parosmia. This knowledge offers promising perspectives for advancing the understanding of the pathophysiology of this condition and for developing novel diagnostic tools and clinical management strategies for patients affected by parosmia.

## Supplementary Information

Below is the link to the electronic supplementary material.

Supplementary file1 (DOCX 56 KB)

## Data Availability

The datasets generated and analysed during the current study are not publicly available due to privacy and ethical restrictions, but are available from the corresponding author on reasonable request and with approval from the institutional ethics committee.
