# Supplementary material for: High sulfur and nitrogen foods are the main triggers of parosmia
Source: Eur Arch Otorhinolaryngol. 2026 May 21;283(7):4477–84. doi: 10.1007/s00405-026-10270-4 (PMC13388635; doi:10.1007/s00405-026-10270-4)
Supplement: Supplementary file 1 — Supplementary file1 (DOCX 56 KB) [file 405_2026_10270_MOESM1_ESM.docx]

**SUPPLEMENTARY INFORMATION 1 Average responses to the Questionnaire of Olfactory Disorders (QOD)**

| **Questions** | **Totally agree** | **Partially agree** | **Partially disagree** | **Torally disagree** |
| --- | --- | --- | --- | --- |
| • The taste of foods is different from what it used to be. | 80 (78,4%) | 19 (18,6%) | 2 (2%) | 1 (1%) |
| • I often perceive a bad smell even when there is no obvious source of odor. | 44 (43,1%) | 28 (27,5%) | 21 (20,6%) | 9 (8,8%) |
| • Other people consider certain odors pleasant that are unpleasant to me. | 88 (86,3%) | 10 (9,8%) | 3 (2,9%) | 1 (1%) |
| • My biggest problem is not that odors are less intense or absent, but that some things smell different than they used to. | 85 (83,3%) | 12 (11,8%) | 2 (2%) | 3 (2,9%) |
| • Because of my olfactory problem, I go to restaurants less often than I used to. | 76 (74,5%) | 13 (12,7%) | 7 (6,9%) | 6 (5,9%) |
| • From the moment I wake up until I go to sleep, I am aware of my difficulties in perceiving odors. | 84 (82,4%) | 16 (15,7%) | 0 (0%) | 2 (2%) |
| • Olfactory difficulties impair my enjoyment of food and beverages. | 91 (89,2%) | 10 (9,8%) | 0 (0%) | 1 (1%) |
| • I am worried I may never be able to cope with this problem. | 67 (65,7%) | 26 (25,5%) | 4 (3,9%) | 5 (4,9%) |
| • I always keep a promise, no matter how difficult it is to fulfill it. | 45 (44,1%) | 39 (38,2%) | 8 (7,8%) | 10 (9,8%) |
| • Due to changes in my olfactory ability, I feel more tense than I used to. | 68 (66,7%) | 24 (23,5%) | 4 (3,9%) | 6 (5,9%) |
| • Sometimes I have thoughts and ideas that I would not want others to know. | 48 (47,1%) | 29 (28,4%) | 14 (13,7%) | 11 (10,8%) |
| • Most of my problems are due to my olfactory difficulties. | 37 (36,3%) | 30 (29,4%) | 20 (19,6%) | 15 (14,7%) |
| • Odor problems bother me while I am eating. | 73 (71,6%) | 21 (20,6%) | 3 (2,9%) | 5 (4,9%) |
| • My behavior is always good and flawless. | 8 (7,8%) | 53 (52%) | 20 (19,6%) | 21 (20,6%) |
| • Because of difficulties with smells, I visit friends, relatives, and neighbors less frequently. | 56 (54,9%) | 15 (14,7%) | 21 (20,6%) | 10 (9,8%) |
| • Because of olfactory difficulties, I struggle to relax. | 45 (44,1%) | 32 (31,4%) | 13 (12,7%) | 12 (11,8%) |
| • Because of difficulties with odors, I have problems with my weight. | 47 (46,1%) | 29 (28,4%) | 18 (17,6%) | 8 (7,8%) |
| • Among all the people I know, there are some I cannot stand being near. | 25 (24,5%) | 27 (26,5%) | 36 (35,3%) | 14 (13,7%) |
| • I can use my imagination to adapt to my olfactory difficulties. | 15 (14,7%) | 34 (33,3%) | 37 (36,3%) | 16 (15,7%) |
| • Olfactory problems make me feel marginalized or isolated. | 34 (33,3%) | 36 (35,3%) | 18 (17,6%) | 14 (13,7%) |
| • Due to odor difficulties, I avoid groups of people. | 28 (27,5%) | 32 (31,4%) | 32 (31,4%) | 10 (9,8%) |
| • Olfactory issues are among life’s problems that one must learn to live with. | 55 (53,9%) | 21 (20,6%) | 19 (18,6%) | 7 (6,9%) |
| • I am never late for appointments or work. | 48 (47,1%) | 29 (28,4%) | 10 (9,8%) | 15 (14,7%) |
| • Because of my smell problems, I eat more or less than I used to. | 74 (72,5%) | 24 (23,5%) | 3 (2,9%) | 1 (1%) |
| • Due to olfactory difficulties, I fear being exposed to certain dangers—gas leaks, spoiled food, etc. | 81 (79,4%) | 11 (10,8%) | 7 (6,9%) | 3 (2,9%) |
| • Due to my smell problems, I have difficulty performing everyday activities. | 31 (30,4%) | 40 (39,2%) | 19 (18,6%) | 12 (11,8%) |
| • Sometimes I talk about things I do not understand. | 14 (13,7%) | 24 (23,5%) | 45 (44,1%) | 19 (18,6%) |
| • My problems with odors make me angry. | 64 (62,7%) | 26 (25,5%) | 6 (5,9%) | 6 (5,9%) |
| • Because of odor problems, my relationship with my spouse is strained. | 23 (22,5%) | 28 (27,5%) | 38 (37,3%) | 13 (12,7%) |
